# Effect of water diuresis with hydrogen saturation on the course of acute kidney damage during the separation of oxidation and phosphorylation

**DOI:** 10.25122/jml-2022-0155

**Published:** 2022-11

**Authors:** Yurii Yevgenivich Rohovyi, Volf Yakovich Tsitrin, Vyacheslav Vasilievich Bilookiy, Michail Ivanovich Sheremet, Oksana Volodimirivna Kolesnik

**Affiliations:** 1 Department of Pathological Physiology, Bukovinian State Medical University, Chernivtsi, Ukraine; 2 Surgery Department No.1, Bukovinian State Medical University, Chernivtsi, Ukraine

**Keywords:** separation of oxidation and phosphorylation, kidney, molecular hydrogen, negative redox potential, CH_2_ONa^+^ – clearance of sodium-free water, DC_c_ – content of diene conjugates in the renal cortex, ENa^+^ – excretion of sodium ions, E_pr_ – excretion of protein, IL-1β – interleukin-1β, IL-6 – interleukin-6, MA_c_– content of malonic aldehyde in the renal cortex, OMP_c_ –content of oxidatively modified proteins in the renal cortex, ORP – oxygen reducing potential, PEM – proton-exchange membrane, PNa^+^ – concentration of sodium ions in blood plasma, ppm – parts per million, ^p^SDA – activity of succinate dehydrogenase in the proximal nephron, RAAS – renin-angiotensin-aldosterone system, SOP – separation of oxidation and phosphorylation, SPE – solid polymer electrolyte, T^d^Na^+^ – reabsorption of sodium ions in the distal nephron, TNFα – tumor necrosis factor-α, UK^+^ – concentrations of potassium ions in urine, UNa^+^ – concentrations of sodium ions in the urine, U_pr_ – concentrations of protein in the urine, URP – redox potential of urine, V– diuresis

## Abstract

Molecular hydrogen has the ability to penetrate cells, easily reach mitochondria, overcome body barriers, penetrate areas of ischemia, edema and inflammation, improve energy supply by supplying additional electrons and have antioxidant and anti-inflammatory effects by neutralizing highly reactive hydroxyl radical and peroxynitrite. In this experiment, we included 60 nonlinear male rats weighing 0.16–0.18 kg and investigated the effect of a negative redox potential solution -297.3±5.27 mV with a molecular hydrogen saturation of 1.2 ppm on the functional-biochemical processes of the kidneys in tissue hypoxia in moderately resistant rats during the separation of oxidation and phosphorylation with the introduction of 2,4-dinitrophenol at a dose of 3 mg/kg. All studies were performed on moderately stable rats. Experimental, functional, biochemical, enzyme-linked immunosorbent, physicochemical, histoenzymochemical, and statistical research methods were used. Under conditions of renal hypoxia in the separation of oxidation and phosphorylation, the use of a solution of negative redox reabsorption of sodium ions in the distal nephron reduces the manifestations of tubular proteinuria, increases the activity of succinate dehydrogenase in the proximal nephron and reduces the redox potential of urine to negative values. Negative redox potential solution with molecular hydrogen saturation has a protective effect on the kidneys and reduces elevated levels of proinflammatory cytokines of tumor necrosis factor-α, interleukin-1-β, and interleukin-6 in blood plasma, and causes oxidative modification of proteins in the renal cortex for their hypoxia in the separation of oxidation and phosphorylation.

## INTRODUCTION

The main rule of kidney energy supply is that the bulk of oxygen absorbed by this organ is used to ensure the reabsorption of sodium ions [1–3]. According to this rule, the kidneys will be particularly responsive to disorders such as tissue hypoxia, which can occur due to the introduction of 2,4-dinitrophenol [1–14]. In tissue hypoxia, the separation of oxidation and phosphorylation (SOP) creates conditions to activate lipid peroxidation, a significant energy deficit of the distal nephron [2, 5]. Hypoxia in the renal tubules is not accompanied by significant inhibitions of the renal blood supply due to SOP. Under these conditions, a sufficient level of glomerular filtration, filtration fraction of sodium ions, and load on the energy-dependent mechanisms of the proximal and distal tubules are likely to be maintained. However, as energy is lacking, there will be significant reactions of proximal nephron damage and distal energy deficiency [1].

Thus, it is important to study the functional, biochemical, enzyme-linked immunosorbent, and histoenzymochemical changes of the kidneys under tissue hypoxia due to the SOP by introducing 2,4-dinitrophenol [15]. In order to correct the detected disorders, it is advisable to use an antioxidant solution of negative redox potential with hydrogen saturation, which has the ability to penetrate cells, easily reach mitochondria, and overcome body barriers: hemato-encephalic, hemato-thyroid, hemato-testicular, hemato-cochlear, hemato-ophthalmic, penetrate areas of ischemia, edema and inflammation, improve energy supply through the supply of additional electrons and exhibit antioxidant action by neutralizing highly reactive hydroxyl radical and peroxynitrite [4, 6, 8]. Considering the facts mentioned above, investigating the mechanisms of a negative redox potential solution with molecular hydrogen saturation on the functional and biochemical processes of the kidneys in tissue hypoxia is relevant and timely. The objective of the study was to determine the effect of a negative redox potential solution with molecular hydrogen saturation of 1.2 ppm on the functional and biochemical processes of the kidneys in tissue hypoxia in moderately resistant rats during SOP.

## MATERIAL AND METHODS

The experiments were performed on 60 nonlinear male rats weighing 0.16-0.18 kg. Prior to the study, all experimental animals were adapted to a hyposodium diet and received food with a low content of sodium ions (wheat grain) and ordinary tap water for 3 days.

Resistance of rats to acute hypoxia was assessed by the time of loss of posture on the “high plateau” of acute hypobaric hypoxia and the time of general stay of animals from the moment of reaching “altitude” 12,000 m to the second agonal breath (lifetime or reserve time) and recovery time from the beginning of the descent. There were 3 groups of animals: high-, medium-, and low-resistant [1]. All studies were performed on moderately stable rats. Simulation of experimental tissue hypoxia was performed by a single intraperitoneal administration of 2,4-dinitrophenol at a dose of 3 mg/kg body weight [2]. The redox potential of water and urine (URP) was determined by an oxygen-reducing potential (ORP) – meter [13]. The effect of loading with ordinary tap water (redox potential 90.4±1.45 mV) the water of negative redox potential with hydrogen saturation 1.0–1.2 ppm and redox potential -297.3±5.27 mV was obtained using a new generation generator Blue Water 900 (Korea), mode 1, which contains an improved proton-exchange membrane PEM/SPE, being also a solid polymer electrolyte [9, 11, 14].

The functional state of the kidneys was studied under a water load. Rats were injected intragastrical with a metal probe using ordinary tap water, water of negative redox potential with additional molecular hydrogen saturation, heated at a temperature of 37℃ in the amount of 5% of body weight. The diuresis (V) amount was estimated in ml/2h × 100 g body weight. After obtaining plasma using water loading, the euthanasia of animals was performed by decapitation under light ether anesthesia, and blood was collected in tubes with heparin. In plasma and urine, the creatinine concentration was determined by reaction with picric acid, sodium and potassium ions – using flame photometry, and protein in urine by the sulfosalicylic method. The excretion of sodium ions (ENa^+^) and protein (E^pr^) was evaluated by the formulas: ENa^+^=V×UNa^+^; E^pr^=V×U^pr^, where UNa^+^ and U^pr^ are the concentrations of sodium ions and protein in the urine, respectively. The clearance of sodium-free (CH_2_ONa^+^) water was calculated by the formula: CH_2_ONa^+^=V-V×UNa^+^/PNa^+^, where PNa^+^ is the concentration of sodium ions in blood plasma. Reabsorption of sodium ions in the distal nephron (T^d^Na^+^) was evaluated by the formula: T^d^Na^+^=(PNa^+^-UNa^+^)×V [7]. Concentrations of sodium and potassium ions in urine (UK+) were determined by flame photometry [3].

The activity of succinate dehydrogenase in the proximal nephron (^p^SDA) of the kidneys was determined by histoenzymochemical method on cryostat sections 10 µm thick using 0.2 M phosphate buffer (pH 7.6), 0.2 M sodium succinate solution and nitrosine tetrazolium. Quantitative analysis of the activity of the studied enzyme was performed by the method of spot test according to Avtandilov G.G. by superimposing templates on the projection of the drug with the subsequent calculation of test points [3]. Enzyme-linked immunosorbent assays were performed using standard kits Amersham (England) and Immuno Nuclear Corporation (USA) to determine the concentration of tumor necrosis factor-α (TNFα), interleukin-1β (IL-1β), interleukin-6 (IL-6). The content of oxidatively modified proteins in the renal cortex (OMP_c_) was determined by reaction with 2,4-dinitrophenylhydrazine to form hydrazones with a characteristic absorption spectrum. The degree of OMP_c_ was judged by the number of formed aldehyde and ketone groups [1]. The content of diene conjugates (DC_c_) and malonic aldehyde (MA_c_) was determined in the renal cortex [3]. According to the statistical analysis of the obtained results, which corresponded to the normal distribution, the methods of variational statistics generally accepted in medicine were used, and the following were calculated: arithmetic mean sample (x) and arithmetic mean standard error (Sx). When estimating the probability of the difference between the average values, the coefficient t was calculated. Reliability (probability of zero hypothesis) for a given t and the number of degrees of freedom was calculated according to the Student's method in a two-way test, and strength (according to an alternative hypothesis) was determined by a one-sided test. To claim the probability of the difference, the standard level of probability p<0.05, generally accepted in bio-biological studies, was considered. We agreed on the use of the Mann-Whitney test. Mann-Whitney (U-test) is a non-parametric statistical test used to assess the difference between two samples in the level of any characteristic measured qualitatively. This test detects differences in the value of a parameter between small samples. In the figures, significantly different values (p<0.05) are marked with the appropriate tags.

## RESULTS

**Figure 1:**
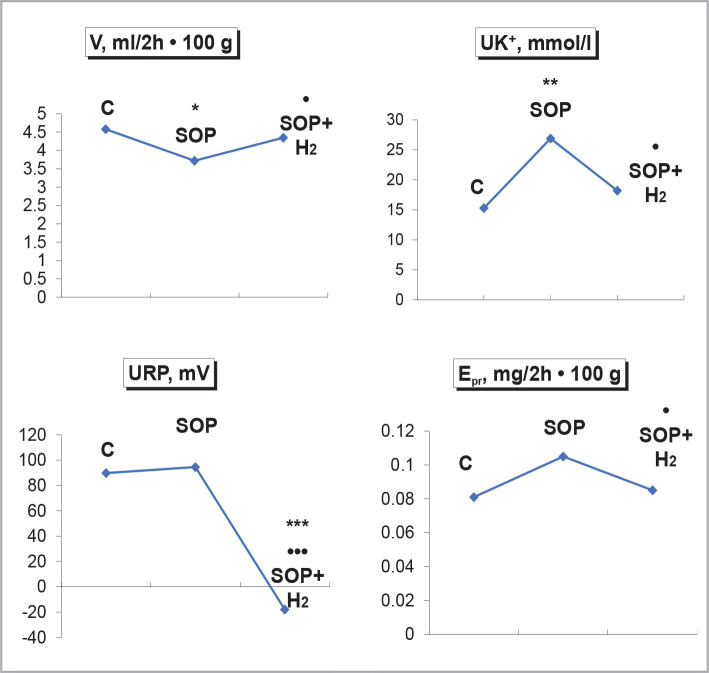
Influence of water load of 5% of body weight with molecular hydrogen saturation 1.2 ppm on diuresis, concentration of potassium ions in urine, redox potential of urine and urinary protein excretion under hypo sodium diet with registration of urine collection for 2 hours during SOP after administration of 2,4-dinitrophenol at a dose of 3 mg kg. Note: here and in Figures 2 and 3. Control (C) – intact animals with a load of ordinary tap water, SOP – SOP when loaded with ordinary tap water, SOP + H_2_ – SOP when loaded with water with molecular hydrogen saturation 1.2 ppm. Significance of differences was noted in comparison with C: * – p<0.05; ** – p<0.02; *** – p<0.01; **** – p<0.001 compared to the SOP: • – p<0.05; •• – p<0.02; ••• – p<0.01.

**Figure 2:**
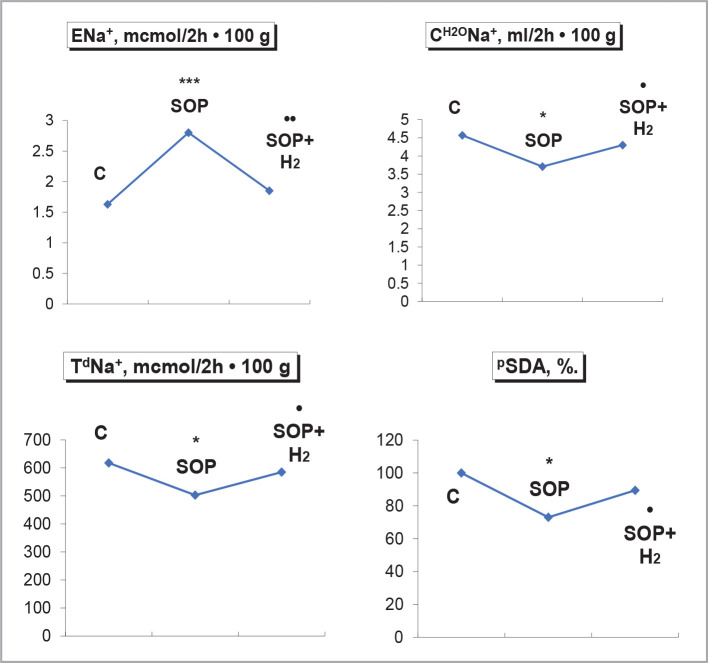
Influence of water load of 5% of body weight with molecular hydrogen saturation of 1.2 ppm on sodium ion excretion, sodium ion free clearance, distal sodium ion reabsorption and succinate dehydrogenase activity in proximal nephron during SOP with the introduction of 2,4-dinitrophenol at a dose of 3 mg/kg.

Hypoxia of the kidneys during SOP was accompanied by inhibition of urinary excretion. An increase in the concentration of potassium ions in the urine and protein excretion was detected. The URP did not change. The use of negative redox fluid with molecular hydrogen saturation increased the level of diuresis, decreased the concentration of potassium ions in the urine, protein excretion, and inhibited the negative redox potential of urine (Figure 1). Under conditions of renal hypoxia due to SOP, there was an increase in excretion of sodium ions, inhibition of clearance of sodium ion-free water, decreased T^d^Na^+^ and inhibition of ^P^SDA. Using a negative redox fluid with molecular hydrogen saturation caused a decrease in the loss of sodium ions in the urine, increased clearance of sodium ion-free water, and increased T^d^Na^+^, ^P^SDA (Figure 2).

**Figure 3:**
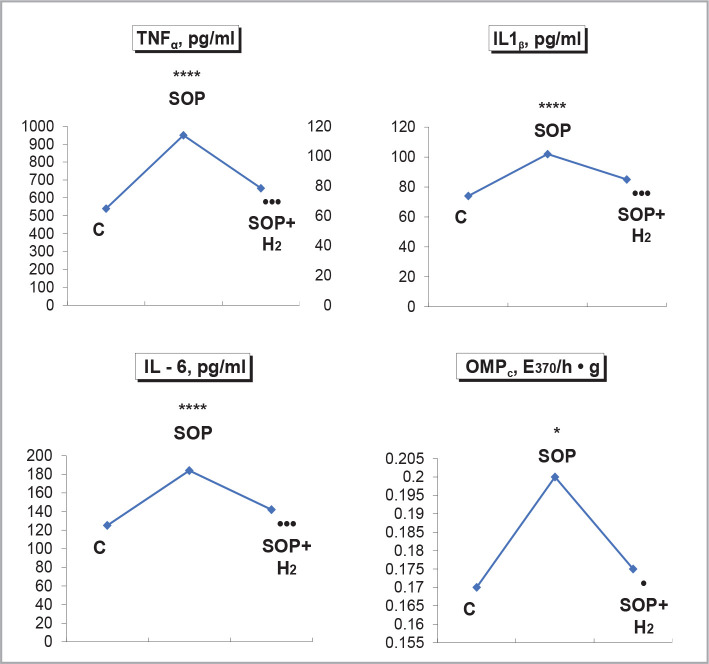
Influence of water load of 5% of body weight with molecular hydrogen saturation 1.2 ppm on the concentration of proinflammatory cytokines in blood plasma and the content of oxidative modification of proteins in the renal cortex of the hypo sodium diet with registration of urine collection for 2h after 2,4-dinitrophenol at a dose of 3 mg/kg.

Under conditions of renal hypoxia due to the SOP, the systemic low-intensity inflammation was activated, as indicated by an increase in plasma concentrations of proinflammatory cytokines: TNFα, IL-1β, IL-6. In the kidneys under these conditions, there was an increase in OMP_c_, MA_c_ by 68.9% (p<0.02), and DC_c_ by 50% (p<0.02). The use of a solution of negative redox potential with molecular hydrogen saturation showed a normalizing anti-inflammatory effect on the state of cytokines: TNFα, IL-1β, and IL-6 (Figure 3). OMP_c_ decreased according to the application of a solution of negative redox potential with molecular hydrogen saturation and also the level of MA_c_ by 21.6% (p<0.05) and DC_c_ by 26.7% (p<0.05).

## DISCUSSION

The introduction of 2,4-dinitrophenol decreased ATP levels in the renal tubules due to the SOP in violation of the main energy-dependent process of the renal tubules – reabsorption of sodium ions, which led to the development of cation loss syndrome. This is confirmed by an increase in the excretion of sodium ions and a decrease in the clearance of sodium-free water. Damage to the intestinal and liver barriers on the background of energy deficiency led to the translocation of endotoxin from the intestinal lumen into the blood, which led to an increase in the concentration of TNFα, which in turn caused increased cytokines IL-1β and IL-6.

**Figure 4:**
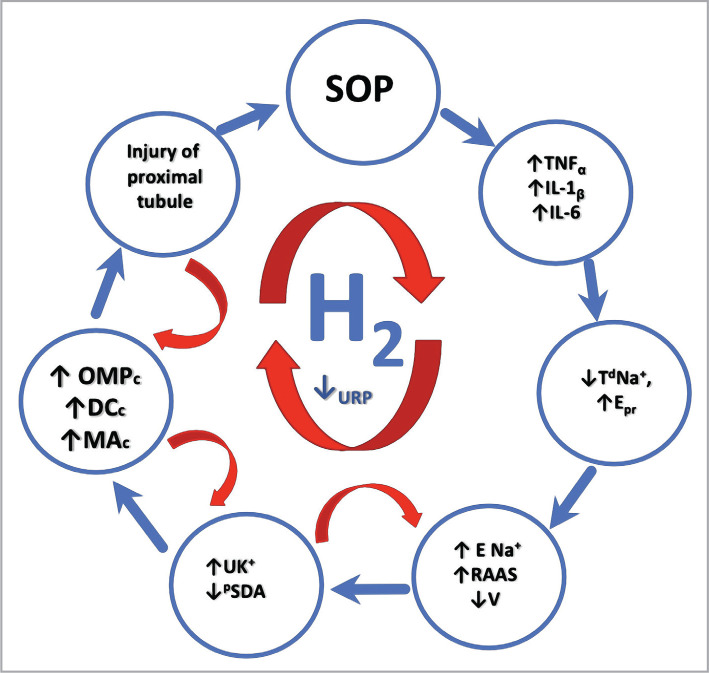
Rupture of large and small vicious circles of proximal nephron damage in tissue hypoxia in moderately resistant rats due in SOP using antioxidant, energy and anti-inflammatory properties of molecular hydrogen (H_2_ [10, 16]). ↑TNF_α_ – increase in the level of TNF_α_ in blood plasma; ↑IL-1β – increase in the level of IL-1β in blood plasma; ↑IL-6 – increase in the level of IL-6 in blood plasma; ↑E_pr_ – increase of protein excretion; ↑ENa^+^ – increase of ENa^+^; ↑UK^+^ – increase concentration of UK^+^; ↑RAAS – activation of RAAS; ↓V – decrease of V; ↓^p^SDA – decrease of ^p^SDA; ↓URP – decrease of URP; ↓T^d^Na^+^ – decrease of T^d^Na^+^; ↑OMP_c_ – increase of OMP in renal cortex; ↑DC_c_ – increase of DC in renal cortex; ↑MA_c_ – increase of MA in renal cortex.

Due to their pro-inflammatory properties, cytokines generated active radicals and additional damage reactions to the renal tubules [16–24] with the increasing manifestation of the syndrome of loss of sodium ions. The points of influence of molecular hydrogen concerning the rupture of a large vicious circle for SOP in rats moderately resistant to hypoxia are based on the results of other studies [25–30] (Figure 4).

SOP leads to growth in the plasma of proinflammatory cytokines of TNFα, IL-1β, and IL-6 (anti-inflammatory effect molecular hydrogen [10, 16]) and causes inhibition of substantially energy-dependent distal reabsorption of sodium ions and development of proximal tubular proteinuria with increasing protein excretion. Furthermore, it leads to urinary sodium ion loss syndrome with activation of the intrarenal renin-angiotensin-aldosterone system (RAAS) with decreased V leading to an increase in the concentration of potassium ions in the urine due to the kaliuretic effect of aldosterone and an increase in the proximal tubule damage with inhibition of ^P^SDA (energy protection effect molecular hydrogen [30]). This leads to further hypoxic activation of lipid peroxidation and OMP_c_, with increasing MA_c_ and DC_c_ (antioxidant effect molecular hydrogen [25–30]) increasing damage to the proximal tubule and the pathogenic effects on the kidneys during SOP. In addition, molecular hydrogen provided the rupture of small vicious circles by the SOP.

Urinary sodium ion loss syndrome with activation of the intrarenal renin-angiotensin-aldosterone system and decreased diuresis led to an increase in the concentration of potassium ions in the urine due to the kaliuretic effect of aldosterone. In addition, it increased damage to the proximal tubule with inhibition of succinate dehydrogenase activity (rupture of the first small vicious circles with molecular hydrogen) [28–30], increasing the syndrome of loss of sodium ions in the urine.

The increase in the concentration of potassium ions in urine due to the kaliuretic effect of aldosterone and the increase in damage to the proximal tubule with inhibition of succinate dehydrogenase activity (rupture of the second small vicious circles due to molecular hydrogen) [25–27] leads to further hypoxic activation of lipid peroxidation in the content of diene conjugates. Furthermore, the increase in the content of malonic aldehyde in the renal cortex leads to further damage to the proximal tubule with inhibition of succinate dehydrogenase activity.

Hypoxic activation of lipid peroxidation and oxidative modification of proteins in the renal cortex with increasing content of diene conjugates and malonic aldehyde in the renal cortex (rupture of the third small vicious circles due to the antioxidant effect of molecular hydrogen) [28] increases damage to the proximal tubule and leads to lipid peroxidation and oxidative modification of proteins in the cortical substance of the kidneys with increasing content of diene conjugates and malonic aldehyde.

## CONCLUSION

Under conditions of renal hypoxia due to SOP, the use of a solution of negative redox potential with molecular hydrogen saturation improves the condition of the distal tubule, reduces the manifestations of proteinuria in the nephron, and reduces the concentration of potassium ions in the urine against the background of reducing the redox potential of urine to negative values. The use of a solution of negative redox potential with molecular hydrogen saturation has a protective effect on the kidneys due to its antioxidant, anti-inflammatory, and nephroprotective properties. It reduces elevated levels of proinflammatory cytokines TNFα, IL-1β, and IL-6 in the blood and oxidative modification of proteins in the cortical substance of the kidneys in their hypoxia due to the SOP.

## References

[1] Belyavskyi VV, Rohovyi YY, Dical MV (2011). Oxidatively modified proteins in rat kidneys and liver in 2,4-dinitrophenol intoxication and melatonin effects in the experiment. Clinical anatomy and operative surgery.

[2] Boychuk VV, Rohovyi YY, Belyavskyi VV, Dical MV (2011). Effect of melatonin on renal function and blood cytokines under the conditions of 2,4-dinitrophenol administration. Herald of Scientific Research.

[3] Boychuk TM, Rohovyi YY, Popovich GB (2012). Pathophysiology of hepatorenal syndrome in hemic hypoxia. Chernivtsi: Medical university.

[4] Chen W, Zhang HT, Qin SC (2021). Neuroprotective Effects of Molecular Hydrogen: A Critical Review. Neurosci Bull.

[5] Honda T, Hirakawa Y, Nangaku M (2019). The role of oxidative stress and hypoxia in renal disease. Kidney Res Clin Pract.

[6] Ishibashi T (2019). Therapeutic efficacy of molecular hydrogen: a new mechanistic insight. Curr Pharm Des.

[7] Krishtal NV, Gojenko AI, Sirman VN (2020). Pathophysiology of the kidneys.

[8] LeBaron TW, Kura B, Kalocayova B, Tribulova N, Slezak J (2019). A New Approach for the Prevention and Treatment of Cardiovascular Disorders. Molecular Hydrogen Significantly Reduces the Effects of Oxidative Stress. Molecules.

[9] Ge L, Yang M, Yang NN, Yin XX, Song WG (2017). Molecular hydrogen: a preventive and therapeutic medical gas for various diseases. Oncotarget.

[10] Nogueira JE, de Deus JL, Amorim MR, Batalhão ME (2020). Inhaled molecular hydrogen attenuates intense acute exercise-induced hippocampal inflammation in sedentary rats. Neurosci Lett.

[11] Ohsawa I, Ishikawa M, Takahashi K, Watanabe M (2007). Hydrogen acts as a therapeutic antioxidant by selectively reducing cytotoxic oxygen radicals. Nat Med.

[12] Rohovyi YY, Bocharov AV, Kobylianska RM (2003). The role of alternative teaching methods in the teaching of theoretical and clinical medical disciplines. Medical education.

[13] Rohovyi YY, Kolesnic OV, Tsitrin VY (2021). Pathophysiology of acute kidney damage with negative redox potential. Chernivtsi: Bukrek.

[14] Rohovyi YY, Tsitrin V, Arkchipova L, Bilookyi V, Kolesnik O (2021). The use of molecular hydrogen in correction of no-reflow syndrome in the polyuric stage of sublimate nephropathy. Georgian Med News.

[15] Seredynska NM, Filipets ND, Filipets OO, Slobodian KV, Hozhenko AI (2019). Experimental models of kidney diseases to study pathogenetic mechanisms and efficacy of pharmacological correction against the background of comorbid pathology. Zapozhye Medical Journal.

[16] Wang B, Li Z, Mao L, Zhao M (2022). Hydrogen: A Novel Treatment Strategy in Kidney Disease. Kidney Dis (Basel).

[17] Alabi QK (2021). Effect of sedentariness on renal function of wistar rats with gentamicin-induced acute kidney injury [dissertacion]. Obafemi Awolowo University;.

[18] Alhusaini AM, Faddah LM, El Orabi NF, Hasan IH (2018). Role of Some Natural Antioxidants in the Modulation of Some Proteins Expressions against Sodium Fluoride-Induced Renal Injury. Biomed Res Int.

[19] Allen JC, Gardner DS, Skinner H, Harvey D (2020). Definition of hourly urine output influences reported incidence and staging of acute kidney injury. BMC Nephrol.

[20] Andreucci M, Faga T, Pisani A, Perticone M, Michael A (2017). The ischemic/nephrotoxic acute kidney injury and the use of renal biomarkers in clinical practice. Eur J Intern Med.

[21] Barasch J, Zager R, Bonventre JV (2017). Acute kidney injury: a problem of definition. Lancet.

[22] Barnett LMA, Cummings BS (2018). Nephrotoxicity and Renal Pathophysiology: A Contemporary Perspective. Toxicol Sci.

[23] Beker BM, Corleto MG, Fieiras C, Musso CG (2018). Novel acute kidney injury biomarkers: their characteristics, utility and concerns. Int Urol Nephrol.

[24] Benoit SW, Devarajan P (2018). Acute kidney injury: emerging pharmacotherapies in current clinical trials. Pediatr Nephrol.

[25] Du H, Sheng M, Wu L, Zhang Y (2016). Hydrogen-Rich Saline Attenuates Acute Kidney Injury After Liver Transplantation via Activating p53-Mediated Autophagy. Transplantation.

[26] Hirano SI, Ichikawa Y, Sato B, Satoh F, Takefuji Y (2020). Hydrogen Is Promising for Medical Applications. Clean Technologies.

[27] Iketani M, Sekimoto K, Igarashi T, Takahashi M (2018). Administration of hydrogen-rich water prevents vascular aging of the aorta in LDL receptor-deficient mice. Sci Rep.

[28] Iuchi K, Nishimaki K, Kamimura N, Ohta S (2019). Molecular hydrogen suppresses free-radical-induced cell death by mitigating fatty acid peroxidation and mitochondrial dysfunction. Can J Physiol Pharmacol.

[29] Jackson K, Dressler N, Ben-Shushan RS, Meerson A (2018). Effects of alkaline-electrolyzed and hydrogen-rich water, in a high-fat-diet nonalcoholic fatty liver disease mouse model. World J Gastroenterol.

[30] Jiang X, Niu X, Guo Q, Dong Y (2019). FoxO1-mediated autophagy plays an important role in the neuroprotective effects of hydrogen in a rat model of vascular dementia. Behav Brain Res.

